# Perceived end-of-life educational needs by clinical trials nurses at a comprehensive cancer center

**DOI:** 10.1016/j.apjon.2022.03.004

**Published:** 2022-03-10

**Authors:** Kristen L. Fessele, Mary Elizabeth Davis, Marlon S. Lasa-Blandon, Maureen E. Reidy, Margaret Barton-Burke

**Affiliations:** a Memorial Sloan Kettering Cancer Center, Office of Nursing Research, New York, NY, USA; b Memorial Sloan Kettering Cancer Center, Evidence Based Practice, New York, NY, USA; c Memorial Sloan Kettering Cancer Center, Clinical Trials Nursing, New York, NY, USA; d Memorial Sloan Kettering Cancer Center, Early Drug Development Service (at Time of Study Conduct), New York, NY, USA

**Keywords:** Palliative care, End-of-life, Cancer, Clinical trials, Nurses, Surveys

## Abstract

**Objective:**

Determine palliative care end-of-life (EOL) educational needs among clinical trials nurses (CTNs) at an urban comprehensive cancer center.

**Methods:**

The End-Of-Life Professional Caregiver Survey (EPCS) was used to determine the EOL educational needs of CTNs and collect demographics on years of experience, education, past EOL-specific training, and possession of their own advanced directive. The “Surprise Question” was also asked to explore the percent of patients on clinical trials who may be nearing EOL.

**Results:**

Twenty-nine CTNs completed the survey. Mean years of experience as an RN and CTN was 10.45 and 2.5, respectively. 79% and 17% held a bachelors or master's degree, respectively. Twenty-seven percent reported previous End-of-Life Nursing Education Consortium (ELNEC) or similar training and 20% stated they had their own advanced directive. Mean total score for the EPCS was 94.83, with subscale means of 42.41 for the Patient and Family Centered Communication (PFCC), 26.9 for Cultural and Ethical Values (CEV), and 25.52 for the Effective Care Delivery (ECD). Highest scoring items included confidence in communicating with colleagues about EOL care, being present with dying patients, and recognizing patients who are appropriate for hospice referral. Lowest scoring items included participating in code status discussions, resolving ethical issues and family conflicts at EOL, and addressing requests for assisted suicide. Responses to the Surprise Question indicated that 27.5% of the CTNs would not be surprised if half or more of their patients died within the next 12 months.

**Conclusions:**

Many patients with cancer on clinical trials may be nearing EOL. CTNs perceive the need for education to increase confidence in handling difficult communication.

## Introduction

Clinical Research Nursing is a specialty practice recognized by the American Nurses Association (ANA) in August 2016. The scope and standards of practice for Clinical Research Nurses (CRN) was developed in collaboration with the International Association of Clinical Research Nurses (IACRN).^1^ The CRN role varies within institutions but usually includes study coordination, eligibility assessment, patient enrolment, patient education or counseling, advocacy for ethical care, trial drug administration, specimen collection, assessment and documentation of toxicity, data management activities, and obtaining or confirming informed consent.^1^, ^2^, ^3^, ^4^ CRN job titles vary and include Study Nurse, Research Nurse Coordinator, Clinical Research Coordinator/Research Assistant, and Clinical Trials Nurse. At the National Cancer Institute (NCI) comprehensive cancer center where this study took place, the term Clinical Trials Nurse (CTN) is used and at the time of writing, there were 146 CTNs involved with 649 active clinical trials across multiple sites for participation (XXX, 2021).

**Fig. 1:**
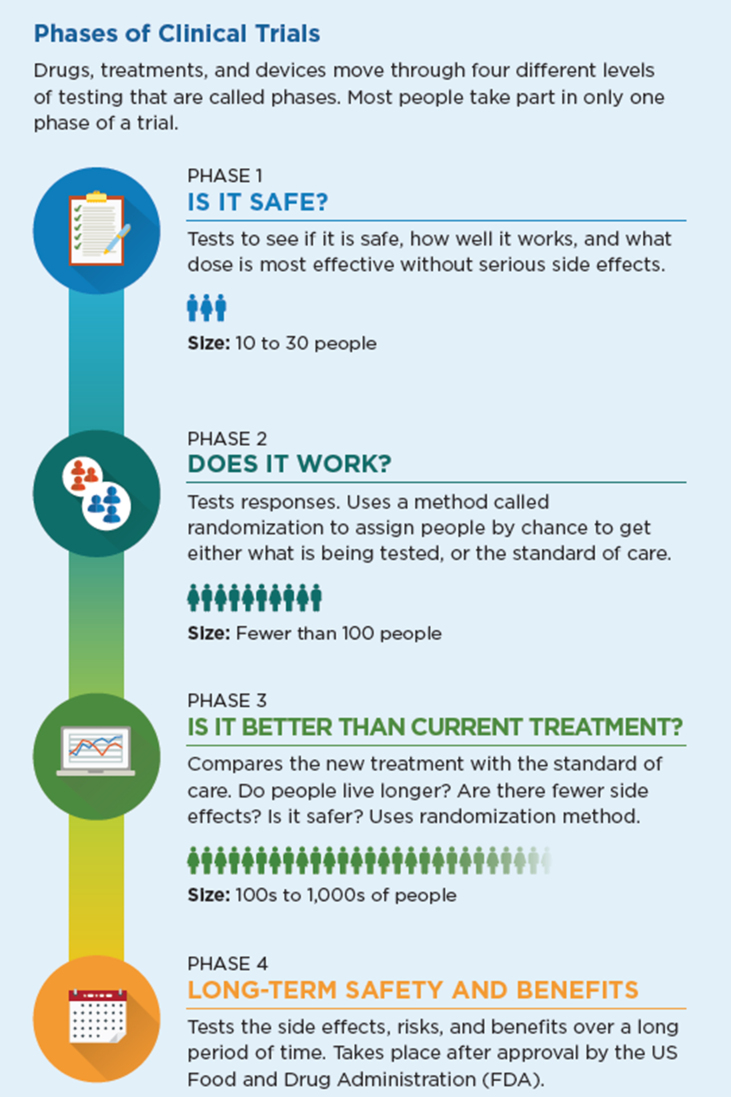
Phases of Clinical Trials. Published with permission by MSK Patient Education Department. Developed with data from the National Cancer Institute^8^

In Fig. 1, the four phases of clinical trials leading to approval of any new drug required by the US Food and Drug Administration (FDA) are illustrated, designed to identify a tolerable dose and major adverse events (Phase I), response rate (Phase II), efficacy compared with current treatments (Phase III) and long term safety (Phase IV).^5^ Since 1992, drugs to treat cancer have often qualified for an accelerated approval process with fewer patients treated per phase, and may be approved by the FDA after meeting a Phase II trial surrogate endpoint such as short-term disease response rate rather than awaiting five year mortality data.^6^, ^7^, ^8^

Also unique to oncology compared with general drug development is ongoing debate regarding the therapeutic intent of a Phase I clinical trial. Most general medicine Phase I trials include healthy volunteers as participants, but patients with advanced cancer who have exhausted other treatment options may be offered the opportunity to receive a drug with a novel mechanism of action but an undetermined dose and adverse event profile.^9^ While most patients remain hopeful for benefit from the treatment,^10^ those who enroll in Phase 1 clinical trials are often in the terminal stage of their disease and have trial response rates ranging from about 3%–10% and overall survival of only 5–9 months.^11^, ^12^, ^13^ A meta-analysis examined perceptions of patients' decisions to participate in cancer clinical trials and found that participation is influenced by the patient perceptions of trust in the clinician, the opinions of their relatives, their assessment of the consequences and benefits of the trial on themself and their family, and finally for altruistic reasons to benefit others.^14^

CTNs working in Phase 1 clinical trials are likely to care for patients presenting with multiple symptoms and complex physical, psychological, spiritual and informational needs.^15^ However, patients on all phases of clinical trials and especially those with progressive disease can benefit from palliative care services. Palliative care is defined as “patient- and family-centered care that optimizes quality of life (QOL) by anticipating, preventing, and treating suffering. Palliative care throughout the continuum of illness involves addressing physical, intellectual, emotional, social, and spiritual needs and to facilitate patient autonomy, access to information, and choice.”^16^ Specialist-led palliative care service models vary and may include consultation and co-management, acute palliative care inpatient units, community-based outreach and hospice coordination.^17^

Primary palliative care includes the provision of basic palliative care performed by the primary clinical team, including CTNs. However, many oncology nurses feel a lack of knowledge of palliative care and concerns communicating with patients and family during end of life care.^15^^,^^18^ Little is known about the preparation and knowledge of CTNs and their perceptions about their ability to provide palliative care in clinical practice. The purpose of this study is to determine the palliative and end-of-life educational needs of CTNs using the End-Of-Life Professional Caregiver Survey (EPCS). Demographic and professional characteristics including years of experience and advanced directives associated with EPCS responses were examined. The results of this study highlight educational opportunities to enhance Clinical Trial Nurses’ preparation and knowledge of palliative and EOL care.

## Methods

### Sample, setting and design

The study population included registered nurses working as CTNs in spring of 2018 ​at an urban multi-site comprehensive cancer center located throughout New York (NY) and New Jersey (NJ). At the time of the study, the department included 94 CTNs and all received the invitation to participate. After obtaining institutional review board approval, the study opportunity was presented at a department staff meeting. Next, an email invitation including a link to the REDCap® data collection platform was sent, allowing CTNs to opt-in to anonymously participate. Two follow up email reminders were sent over the three-week data collection period to maximize response.

### Instruments

The main study instrument was the EPCS^19^, a 28-item questionnaire that uses a five-point Likert scale to assess educational needs across professions. Higher scores reflect greater perceived skills or availability of resources to engage in EOL care, with responses for each item ranging from “not at all” to “very much.” It was developed based on eight domains of palliative and EOL care that align with national physician and nursing core curricula^20^^,^^21^: scientific and clinical knowledge/technical skills; communication/interpersonal skills with patients, family members, and other clinicians; spiritual and cultural issues; ethical, professional, and legal principles; organizational skills; and attitudes, values, and feelings of health care professionals. The survey authors determined reliability statistics using Cronbach's α ​> ​0.70 as evidence of adequate scale reliability. The tool further revealed three factors leading to development of subscales, including the Patient- and Family-Centered Communication (PFCC), Cultural and Ethical Values (CEV) and Effective Care Delivery (ECD) components of the instrument.^22^ Feder et al^23^ reported Cronbach's α of EPCS as 0.96 for all items, 0.95 for PFCC, 0.89 for CEV and 0.87 for ECD. The EPCS has been used in national and international studies and has been validated in several languages.^22^, ^23^, ^24^ Possible total EPCS scores range from 28 to 140, with the PFCC subset range from 12 to 60, and the CEV and ECD subsets from 8 to 40.

Demographic information on education, years of experience as an RN and as a clinical trials nurse and possession of their own advanced directive was collected. Previous EOL training was recorded as participation in the End of Life Nursing Education Consortium (ELNEC, a palliative care core curriculum developed in 2000 by City of Hope, Duarte, CA and the American Association of Colleges of Nursing (AACN^21^), Hospice and Palliative Care Certification (CHPN^25^) or any EOL training course greater than 4 ​h in duration. As an exploratory item, the “Surprise Question,” a single item query that has been used as a trigger for referral to palliative care in dialysis and cancer populations^26^, ^27^, ^28^ was asked. A “no” response by the clinician to the question “Would I be surprised if this patient died in the next year?’’ identified patients with cancer who had a seven-fold higher likelihood of one-year mortality (HR=7.78, *p* ​< ​0.001^29^). A more recent systematic review of 26 articles across multiple palliative populations found a wide positive predictive validity range from 13.9% to 78.6% with overall accuracy of approximately 75%.^30^ For clarity in this study setting, the question was phrased as, “What percent of your patients do you estimate might die within the next year?”

Data were analyzed in SPSS version 26. The means and standard deviation were computed for the total EPCS scores and the PFCC, CEV. and ECC subscales and associations between years of experience, possession of their own advanced directive or prior EOL training and EPCS scoring were analyzed.

## Results

Forty-five CTNs at least partially responded to the survey, with a total of 29 who completed all items ( Table 1). Mean years as a registered nurse was 10.45 (range 2–35 years) with an average of 2.48 years as a CTN (1–10 years). Seventy-nine percent and 17% reported their highest degree as a bachelor's or master's degree, respectively. 75% were assigned to the institution's main locations in XXX, with the remainder at other regional centers in either XXX or XXX. Five CTNs (17%) reported previous ELNEC training and three (10%) reported similar past training. Six (20%) stated they had their own advanced directive.

**Table 1:** Characteristics of Participants (*n* ​= ​29)

	*M*	*n*	*%*
Years as a registered nurse^1^	10.45		
≤5		9	31.0
6–9		6	20.7
10–12		7	24.2
≥13		7	24.1
Years as a clinical trials nurse	2.48		
≤1		17	58.6
2–4		6	20.6
5–7		5	17.2
≥8		1	3.4
Highest level of education			
Bachelors		23	79.3
Masters		5	17.2
Missing		1	3.4
Work location			
∗Blinded for review∗		22	75.9
Regional location		7	24.1
Had previous end of life training			
No		21	72.4
ELNEC		5	17.2
HPCN		0	0
Similar course >4 ​h in length		3	10.3
Has own advance directive			
Yes		6	20.7
No		20	69.0
Prefer not to answer		1	3.4
Missing		2	6.9

ELNEC, End-of-Life Nursing Education Consortium; HPCN, Hampshire Parent Carer Network.

**Fig. 2:**
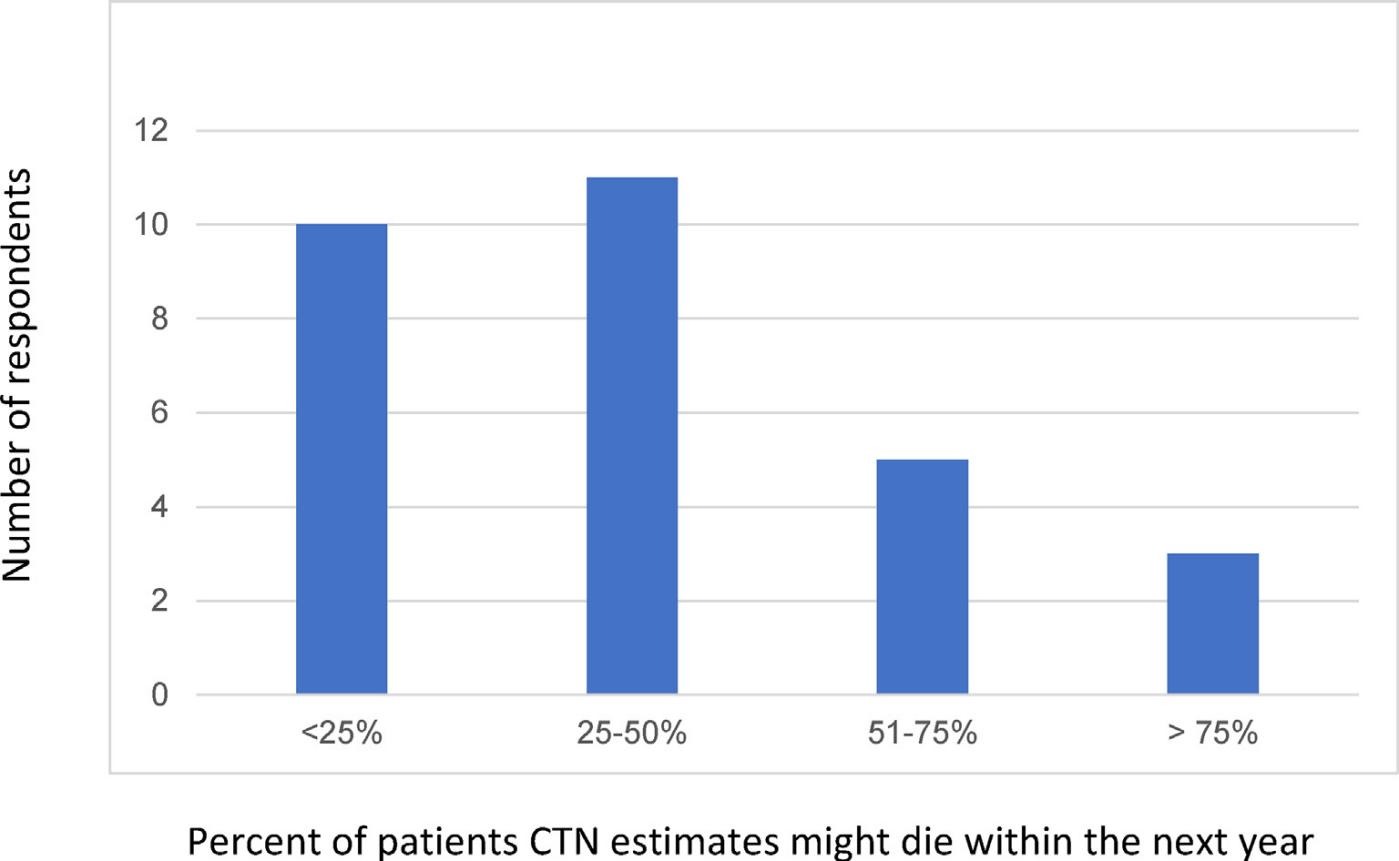
“Surprise Question” Responses. The question item was phrased as “What percent of your patients do you estimate might die within the next year?”

Mean total score for the CTN sample on the EPCS was 94.83 (SD 20.26). Subscale scores included a mean of 42.41 (SD 8.19) for the PFCC, 26.9 (SD 6.82) for CEV and 25.52 (SD 6.98) for the ECD. There were no significant differences in scores when controlling for prior EOL training, degree, years of experience or possession of an advanced directive. Item means within each subscale varied (Table 2). In the PFCC, the lowest scoring items, “I am comfortable starting and participating in discussions about code status” and “I am comfortable helping to resolve difficult family conflicts about end-of-life care” scored a mean of 2.76 (SD 1.4 and 1.27, respectively) with “I am comfortable talking with other health care professionals about the care of dying patients” scoring highest at a mean of 4.21 (SD 0.675). The lowest mean score in the CED subscale was “I am comfortable dealing with ethical issues related to end-of-life/hospice/palliative care” at 3.0 (SD 1.03) with highest of 3.79 (SD 0.978) for “I am able to be present with dying patients.” In the ECD subscale, the lowest scoring item mean was “I feel confident addressing requests for assisted suicide” at 2.17 (SD 1.42) and highest at “I can recognize when patients are appropriate for referral to hospice” at 3.69 (SD 0.81). In answer to the “Surprise Question” asking what percentage of patients in their caseload they estimate might die within the next year, 34.5% (*n* ​= ​10) of respondents noted they would not be surprised if a quarter or fewer died, 38% (*n* ​= ​11) felt 25%–50% might die, 17.2% (*n* ​= ​5) expected 50%–75% could die and 10.3% (*n* ​= ​3) believed that more than 75% of their patients might not live more than a year (Fig. 2).

**Table 2:** EPCS Item Responses by Subscale

Item	*Mean(SD)*
EPCS Total Score	94.8 (20.3)
**Patient and Family Centered Communication (PFCC) Total Subscale Score**	42.4 (8.2)
1. I am comfortable helping families to accept a poor prognosis	3.38 (1)
2. I am able to set goals for care with patients and families	3.41 (1.1)
3. I am comfortable talking to patients and families about personal choice and self-determination	3.52 (0.91)
4. I am comfortable starting and participating in discussions about code status	2.76 (1.4)
5. I can assist family members and others through the grieving process	3.59 (1)
6. I am able to document the needs and interventions of my patients	4.17 (0.66)
7. I am comfortable talking with other health care professionals about the care of dying patients	4.21 (0.68)
8. I am comfortable helping to resolve difficult family conflicts about end-of-life care	2.76 (1.3)
9. I can recognize impending death (physiologic changes)	4.14 (0.64)
10. I know how to use nondrug therapies in management of patients' symptoms	3.55 (0.91)
11. I am able to address patients' and family members' fears of getting addicted to pain medications	3.62 (0.86)
12. I encourage patients and families to complete advance care planning	3.31 (1.3)
**Cultural and Ethical Values (CEV) Total Subscale Score**	26.9 (6.8)
13. I am comfortable dealing with ethical issues related to end-of-life/hospice/palliative care	3.00 (1)
14. I am able to deal with my feelings related to working with dying patients	3.69 (0.76)
15. I am able to be present with dying patients	3.79 (0.98)
16. I can address spiritual issues with patients and their families	3.24 (1.2)
17. I am comfortable dealing with patients' and families' religious and cultural perspectives	3.62 (1)
18. I am comfortable providing grief counseling for families	3.17 (1.2)
19. I am comfortable providing grief counseling for staff	3.14 (1.1)
20. I am knowledgeable about cultural factors influencing end-of-life care	3.24 (1)
**Effective Care Delivery (ECD) Total Subscale Score**	25.5 (7.0)
21. I can recognize when patients are appropriate for referral to hospice	3.69 (0.81)
22. I am familiar with palliative care principles and national guidelines	3.28 (1.1)
23. I am effective at helping patients and families navigate the health care system	3.34 (1)
24. I am familiar with the services hospice provides	3.48 (0.87)
25. I am effective at helping to maintain continuity across care settings	3.52 (1)
26. I feel confident addressing requests for assisted suicide	2.17 (1.4)
27. I have personal resources to help meet my needs when working with dying patients and families	3.03 (1.4)
28. I feel that my workplace provides resources to support staff who care for dying patients	3 (1.2)

EPCS, ​End-of-Life Professional Caregiver Survey.

## Discussion

This study examined perceived palliative and end-of-life care educational needs among 29 oncology clinical trials nurses at an urban, NCI-designated cancer center using the EPCS. The CTN sample in this study demonstrated a lower mean overall score of 94.83 compared with other reports (107.7 in Lazenby's 2012 study^19^ and 106.6 in Feder's validation among Brazilian nurses^23^), indicating a strong opportunity for focused palliative care education. Subscale mean scores were also notably lower for the PFCC in our CTN sample compared with Lazenby and Feder's samples, respectively (42.41 vs. 47.1 and 48.4), 26.9 for CEV (compared with 29.1 and 31.4) and 25.52 vs. 29.2 and 26.8 for the ECD.

Noting the responses in this sample within each subscale, EPCS items with the highest scores seemed to be related to concrete nursing skills with lower scores specific to items that required more advanced EOL communication expertise. For example, in the PFCC subscale the highest scoring items were related to talking to other health care providers (*M* ​= ​4.21), documenting needs (*M* ​= ​4.17), and recognizing physical signs of impending death (*M* ​= ​4.14). Talking with the patient and family about code status (*M* ​= ​2.76), advance care planning (*M* ​= ​3.31) and resolving family conflicts (*M* ​= ​2.76) were the lowest scoring PFCC items. This aligns with Toh et al^18^ who found nurses experienced difficulties with communication when providing EOL care. Ben-Zacharia et al also found significant association between palliative care/EOL training and comfort in discussing code status and advanced directives with patients and families.^31^

In the CEV subscale, the highest scoring items included being present with the dying (*M* ​= ​3.79), dealing with own feelings about working with the dying (*M* ​= ​3.69), dealing with religious and cultural perspectives (*M* ​= ​3.62). The lowest scores related to providing grief counseling to staff or families (*M* ​= ​3.17) and addressing spiritual issues (*M* ​= ​3.24). Responses in the ECD subscale indicate participants were most comfortable recognizing when a patient is an appropriate referral candidate to hospice (*M* ​= ​3.69) and familiarity with hospice services (*M* ​= ​3.48) and least confident addressing requests for assisted suicide (*M* ​= ​2.17). The latter result is consistent with findings from Lazenby's 2012 study^19^ and could be related to lack of experience as assisted suicide is not legal in XXX where the majority of participating CTNs (75.9%) practiced.

While multiple studies showed a relationship between a health care provider having a personal advanced directive and higher EPCS scores,^19^^,^^31^^,^^32^ and higher scores with greater years of experience and education,^19^^,^^23^^,^^31^^,^^33^, ^34^, ^35^ this analysis did not. This may be due to the relatively small, less clinically experienced sample in this study and was consistent with EPCS results in a Brazilian sample of palliative care nurses^34^ and among medical/surgical and intensive care unit nurses in a North Carolina hospital.^36^

This study has some notable limitations. To promote confidentiality given the characteristics of the staff at the time of survey, the respondents were not asked to report their age or gender. The current role of the CTN at this cancer institute was introduced in 2016. Prior to this, the role was identified as “research nurse” and responsibilities varied from that of the CTN today. When asked about the number of years as a CTN, the survey did not specify, if applicable, to include the number of years practicing under the retired title of research nurse, nor did it instruct the participant to provide only the number of years practicing as a CTN at this institution or include previous healthcare settings. In addition, the participants were not asked if they were an advance practice provider (physician assistant, nurse practitioner).

Originally, the intended focus of this study was on CTNs providing care to patients with the most advanced disease and poorest prognosis, such as the Phase I patient population. Due to the small size of that team, the choice was made to survey the full CTN department. It was not possible to use phase of clinical trial as a proxy for patient status as CTNs outside the Phase I team provide care for patients participating in multiple phases of clinical trials. Instead, this study explored use of the Surprise Question and found that CTNs believed they cared for patients with very advanced disease. Almost two thirds of participants would not be surprised if 25% or more of their patients did not live more than a year.

## Conclusions

To provide the highest quality care to patients with cancer, there is consensus that palliative care must be integrated across care settings and disciplines.^10^ Primary palliative care education allows oncology clinicians not part of a specialized consultative team to provide the essential components of palliative care to any patients in their caseload who require it. New models of care are under investigation on how to best disseminate primary palliative care balanced with support from consultative palliative specialists.^6^^,^^10^ Patients on clinical trials are a vulnerable population, often with advanced or rare cancers and need a great deal of support and resources from their health care providers. Specialized education such as the ELNEC and other EOL training program equip clinicians to help patients be listened to, heard and have their burden acknowledged.^11^ In addition to didactic content, the lowest scoring EPCS items in this study related to talking with patients and family about difficult EOL topics, indicating a strong need for oncology nurses to practice advanced communication skills. Role play and other simulation approaches with “standardized” participants (i.e. actors trained to portray patient and family member responses during a training simulation) can be extremely effective methods to increase communication skills for nurses.^37^, ^38^, ^39^ Strong communication and advanced care planning skills support nurses to deeply engage with patients to contribute to their plan of care during active treatment and at the end of their lives.

These results contribute to the understanding of the palliative education needs of CTNs. The subscales of the EPCS map to components of the ELNEC curriculum, which will facilitate future efforts to provide tailored education at this institution as one outcome of this study. Future work is needed to explore EPCS scoring across other nursing teams and disciplines.

## References

[1] Association A.N. (2016).

[2] Herzog-LeBoeuf C., Willenberg K.M. (2020). The history of clinical trials research: implications for oncology nurses. Semin Oncol Nurs.

[3] Ness E.A., Royce C. (2017). Clinical trials and the role of the oncology clinical trials nurse. Nurs Clin.

[4] McCabe M., Ness E. (2021). Margaret McCabe Elizabeth Ness EBook : Elizabeth Ness, Editors Margaret McCabe: Kindle Store. International Association of Clinical Research Nurses.

[5] FDA (2018). https://www.fda.gov/patients/drug-development-process/step-3-clinical-research.

[6] Gyawali B., Hey S.P., Kesselheim A.S. (2019). Assessment of the clinical benefit of cancer drugs receiving accelerated approval. JAMA Intern Med.

[7] Chabner B. (2012). Approval of new agents after phase II trials. Am Soc Clin Oncol Educ B.

[8] Phases of Cancer Clinical Trials - National Cancer Institute (2020). https://www.cancer.gov/about-cancer/treatment/clinical-trials/what-are-trials/phases.

[9] Tao D.L., Kartika T., Tran A., Prasad V. (2020). Phase I trials and therapeutic intent in the age of precision oncology: what is a patient's chance of response?. Eur J Cancer.

[10] Ferrell B.R., Chung V., Koczywas M., Smith T.J. (2020). Dissemination and implementation of palliative care in oncology. J Clin Oncol.

[11] Rezash V., Reed J., Gedeon B. (2020). Who needs what? Perceptions of patients and caregivers in oncology phase 1 trials. J Patient Exp.

[12] Godskesen T., Nygren P., Nordin K., Hansson M., Kihlbom U. (2013). Phase 1 clinical trials in end-stage cancer: patient understanding of trial premises and motives for participation. Support Care Cancer.

[13] Schupmann W., Miner S.A., Sullivan H.K. (2021). Exploring the motivations of research participants who chose not to learn medically actionable secondary genetic findings about themselves. Genet Med.

[14] Nielsen Z.E., Berthelsen C.B. (2019). Cancer patients' perceptions of factors influencing their decisions on participation in clinical drug trials: a qualitative meta-synthesis. J Clin Nurs.

[15] Parajuli J., Hupcey J.E., Kitko L., Birriel B. (2021). Palliative care: oncology nurses' confidence in provision to patients with cancer. Clin J Oncol Nurs.

[16] (2021). Palliative care overview | NHPCO.

[17] Hui D., Bruera E. (2020). Models of palliative care Delivery for patients with cancer. J Clin Oncol.

[18] Toh S.W., Hollen V.T., Ang E., Lee Y.M., Devi M.K. (2021). Nurses' communication difficulties when providing end-of-life care in the oncology setting: a cross-sectional study. Support Care Cancer.

[19] Lazenby M., Ercolano E., Schulman-Green D., McCorkle R. (2012). Validity of the end-of-life professional caregiver survey to assess for multidisciplinary educational needs. J Palliat Med.

[20] (2021). Home: EPEC: Education in Palliative and End-Of-Life Care: Feinberg School of Medicine.

[21] (2021). End-of-Life-Care (ELNEC). https://www.aacnnursing.org/ELNEC.

[22] Cláudia Mesquita Garcia A., Marina Calixto Damasceno Spineli V., Helena Appoloni Eduardo A., Meireles E., Antonio Moreira de Barros G., Lazenby M. (2019). Translation, cultural adaptation, and validation of the Brazilian Portuguese version of the end-of-life professional caregiver survey. Palliat Support Care.

[23] Feder S.L., Collett D., Conley S., Schulman-Green D., Meron T., Cherny N. (2018). How skilled do Israeli nurses perceive themselves to be in providing palliative care? Results of a national survey. Int J Palliat Nurs.

[24] Zou Z., Bai J., Gu Y. (2021). Cultural adaptation and psychometric evaluation of the Chinese version of the nurse-specific end-of-life professional caregiver survey: a cross-sectional study. BMC Palliat Care.

[25] (2021). CHPN.

[26] Murray S.A., Boyd K. (2011). Using the’surprise question’can identify people with advanced heart failure and COPD who would benefit from a palliative care approach. Palliat Med.

[27] Downar J., Goldman R., Pinto R., Englesakis M., Adhikari N.K.J. (2017). The “surprise question” for predicting death in seriously ill patients: a systematic review and meta-analysis. CMAJ (Can Med Assoc J).

[28] Hudson K.E., Wolf S.P., Samsa G.P., Kamal A.H., Abernethy A.P., LeBlanc T.W. (2018). The surprise question and identification of palliative care needs among hospitalized patients with advanced hematologic or solid malignancies. J Palliat Med.

[29] Moss A.H., Lunney J.R., Culp S. (2010). https://home.liebertpub.com/jpm.

[30] White N., Kupeli N., Vickerstaff V., Stone P. (2017). How accurate is the ‘Surprise Question’at identifying patients at the end of life? A systematic review and meta-analysis. BMC Med.

[31] Ben-Zacharia A.B., Bethoux F.A., Volandes A. (2021). Self-perceived knowledge and comfort discussing palliative care and end-of-life issues among professionals managing neuroinflammatory diseases. J Palliat Med.

[32] Wallace C.L., Cruz-Oliver D.M., Ohs J.E., Hinyard L. (2018). Connecting personal experiences of loss and professional practices in advance care planning and end-of-life care: a survey of providers. Am J Hospice Palliat Med.

[33] Moir C., Roberts R., Martz K., Perry J., Tivis L. (2015). Communicating with patients and their families about palliative and end-of-life care: comfort and educational needs of nurses. Int J Palliat Nurs.

[34] Garcia A.C.M., Damasceno Spineli V.M.C., Eduardo A.H.A., Meireles E., Moreira De Barros G.A., Lazenby M. (2020). Translation, cultural adaptation, and validation of the Brazilian Portuguese version of the end-of-life professional caregiver survey. Palliat Support Care.

[35] O'Shea E.R., Lavallee M., Doyle E.A., Moss K. (2017). Assessing palliative and end-of-life educational needs of pediatric health care professionals: results of a statewide survey. J Hospice Palliat Nurs.

[36] Mason M. (2018).

[37] Coyle N., Manna R., Shen M. (2015). Discussing death, dying, and end-of-life goals of care: a communication skills training module for oncology nurses. Clin J Oncol Nurs.

[38] Banerjee S.C., Manna R., Coyle N. (2017). The implementation and evaluation of a communication skills training program for oncology nurses. Transl Behav Med.

[39] Zavertnik J.E., Huff T.A., Munro C.L. (2010). Innovative approach to teaching communication skills to nursing students. J Nurs Educ.

